# Hidden talents in harsh conditions? A preregistered study of memory and reasoning about social dominance

**DOI:** 10.1111/desc.12835

**Published:** 2019-05-16

**Authors:** Willem E. Frankenhuis, Sarah A. de Vries, JeanMarie Bianchi, Bruce J. Ellis

**Affiliations:** ^1^ Behavioural Science Institute, Radboud University Nijmegen The Netherlands; ^2^ Institute of Cognitive and Evolutionary Anthropology University of Oxford Oxford United Kingdom; ^3^ Department of Psychology The University of Arizona Tucson Arizona; ^4^ Departments of Psychology and Anthropology University of Utah Salt Lake City Utah

## Abstract

Although growing up in stressful conditions can undermine mental abilities, people in harsh environments may develop intact, or even *enhanced*, social and cognitive abilities for solving problems in high‐adversity contexts (i.e. ‘hidden talents’). We examine whether childhood and current exposure to violence are associated with memory (number of learning rounds needed to memorize relations between items) and reasoning performance (accuracy in deducing a novel relation) on transitive inference tasks involving both violence‐relevant and violence‐neutral social information (social dominance vs. chronological age). We hypothesized that individuals who had more exposure to violence would perform better than individuals with less exposure on the social dominance task. We tested this hypothesis in a preregistered study in 100 Dutch college students and 99 Dutch community participants. We found that more exposure to violence was associated with lower overall memory performance, but not with reasoning performance. However, the main effects of current (but not childhood) exposure to violence on memory were qualified by significant interaction effects. More current exposure to neighborhood violence was associated with worse memory for age relations, but not with memory for dominance relations. By contrast, more current personal involvement in violence was associated with *better* memory for dominance relations, but not with memory for age relations. These results suggest incomplete transfer of learning and memory abilities across contents. This pattern of results, which supports a combination of deficits and ‘hidden talents,’ is striking in relation to the broader developmental literature, which has nearly exclusively reported deficits in people from harsh conditions. A video abstract of this article can be viewed at: https://youtu.be/e4ePmSzZsuc.

## Graphical abstract

People who grow up in stressful conditions may develop ‘hidden talents’: intact, or even enhanced, abilities for solving problems in high‐adversity contexts. We find partial support for this hypothesis. In our study, more current exposure to violence predicts intact or even enhanced memory for social dominance relationships, but more childhood exposure to violence predicts impaired memory for social dominance relationships.

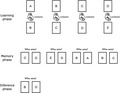

**Research Highlights**

- People who grow up in stressful conditions may develop ‘hidden talents’: intact, or even *enhanced*, abilities for solving problems in high‐adversity contexts.
- Contrary to this hypothesis, in our study *childhood* exposure to violence was negatively or not associated with memory and inference for social dominance content.
- However, *current* exposure to violence was associated with equal, or even better, memory performance (but not reasoning) for social dominance content.
- Our data thus provide some support for the existence of a ‘hidden talent’, in addition to evidence for associations between adversity and impairment.

## 1 **INTRODUCTION**

It is well‐established that growing up under conditions of social and economic adversity can undermine children's development, cognitive abilities, and health (Duncan, Magnuson, & Votruba‐Drzal, 2017; Nelson, Fox, & Zeanah, 2014). An extensive body of research has demonstrated these harmful effects, producing valuable knowledge that has informed educational and economic policies and interventions designed to prevent and repair deficits. Far less recognized is an emerging body of research showing that people from high‐adversity backgrounds may develop enhanced social and cognitive abilities that are adapted to stressful conditions (known as the ‘specialization hypothesis’; Ellis, Bianchi, Griskevicius, & Frankenhuis, 2017; Frankenhuis & de Weerth, 2013). Documenting these ‘hidden talents’—and then redesigning teaching, learning, and assessment processes to capitalize on these abilities in school—is a potential tool for improving the academic performance of students from socially and economically disadvantaged backgrounds. Using these abilities as building blocks for success may boost confidence, motivation, and hence performance in people who suffer from stigma and hardship. Thus, to complement the prevailing focus on deficits, we need to identify the abilities that are enhanced by chronic exposures to stress. In the future, we envision an assessment battery that measures these hidden talents, which can be used to benefit high‐adversity youth in education, jobs, and civic life (Ellis et al., 2017).

This ‘hidden talents’ approach is based on an evolutionary‐developmental perspective focusing on adaptation in context (Ellis et al., 2017; Frankenhuis & de Weerth, 2013). It dovetails with research in cultural psychology, which regards differences in performance between individuals and social groups—including different socioeconomic groups—as not necessarily resulting from deficits, but as potentially resulting from people using their intelligence to solve locally significant challenges within the constraints posed by their environments (Banerjee, Bhattacharjee, Chattopadhyay, & Ganimian, 2017; Greenfield, 2014; Grossmann & Varnum, 2011; Kraus, Piff, Mendoza‐Denton, Rheinschmidt, & Keltner, 2012; Rogoff et al., 2017; Schliemann & Carraher, 2002; Sternberg, 2014; Varnum & Kitayama, 2017). Social and economic adversity can be regarded as one context that gives rise to specific knowledge, skills, and abilities. Such stress‐adapted skills, however, may be less likely to manifest in evolutionarily novel contexts, such as formal classroom settings, than in the ecological contexts in which they developed, such as neighborhood and family settings. Further, there are multiple dimensions of childhood stress (e.g. neglect, violence, unpredictability), each of which poses unique challenges that may promote the development of particular skills and abilities. For this reason, empirical studies of ‘hidden talents’ may benefit from measuring exposures to specific dimensions of stress, rather than general social or economic adversity (e.g. cumulative stress scores), which do not capture more fine‐grained individual variation.

### 1.1 Memory and reasoning about social dominance versus chronological age

The current study focuses on the challenge of navigating social hierarchies in violent environments. Social status is a crucial factor that determines access to resources in both humans and non‐human animals (Hawley, 1999). In order to respond effectively to potential rivals, allies, and mates, individuals need to determine their own and others’ social status (Penn, Holyoak, & Povinelli, 2008). Inferring social rank from observations of interactions is a vital skill, as direct confrontations can be time‐consuming and entail the risk of injury or death (Grosenick, Clement, & Fernald, 2007; Nakamaru & Sasaki, 2003; Paz‐y‐Miño, Bond, Kamil, & Balda, 2004; Wynne, 1995). Humans infer social dominance even from thin slices of behavior (Ambady, Bernieri, & Richeson, 2000).

Social hierarchies are often linear (Fiske, 1992; Gazes, Hampton, & Lourenco, 2015; Hawley, 1999; Hsu, Earley, & Wolf, 2006; De Soto, 1960; Zitek & Tiedens, 2012). Therefore, dominance relations may be inferred using transitive inference (TI): the ability to infer unknown relations between items (e.g. A > C), based on known relations of those items (e.g. A > B; B > C). TI involves memory of premises (e.g., A > B; B > C) and inference of a conclusion (e.g. A > C). Variation in performance may result from differences in memory, inference, or both. Hence, it is crucial to study the separate contributions of these processes (Bryant & Trabasso, 1971; Chalmers & McGonigle, 1984; Wright, 2012).

We theorized that inferring social dominance—in particular, forms of social dominance that involve the ability to win in a physical fight—is a critical task for people from harsh‐dangerous environments. In such contexts, the costs of incorrect memories and inferences about dominance are likely to be greater than in safer environments. For instance, in violent street gangs, challenging a dominant individual could trigger a beating or deadly assault (Keiser, 1979; Shakur, 2007; Venkatesh, 2008). Therefore, *we hypothesized that people who have had greater exposure to violence develop enhanced memory and reasoning for dominance relations, compared with people who have had less exposure to violence*. Our task stimuli specifically focused on dominance relations among young adult men. We operationalized exposure to violence both in terms of the amount of violence in one's lived environment (e.g. perceived neighborhood violence, witnessing fights) and one's direct involvement in physical fighting (e.g. frequency of involvement, severity of injuries).

To test our hypothesis, we examined memory and reasoning performance across two different relational domains: social dominance and chronological age. We included age relations, which are commonly included in conventional TI problems (Burt, 1919; Markovits & Dumas, 1999; Piaget, 1955), as a comparison variable. We expected small age differences to be equally (ir)relevant to all participants, regardless of exposure to violence. Although age can provide cues to rates of violence (e.g. young men fight more), age cues are not informative if age differences are small, as they were in our stimuli (i.e. all young adult men). We conceptualized minor age variations as exemplars of the kind of abstract or irrelevant information that tends to undermine performance on cognitive tasks among people from high‐adversity backgrounds (see Ellis et al., 2017). Because performance on conventional TI tasks is positively correlated with IQ (Burt, 1919; Sternberg, 1983), and because people exposed to social and economic adversity tend to score lower on IQ tests (APA Task Force on Socioeconomic Status, 2007), exposure to harsh environments may be associated with lower performance on conventional TI problems involving abstract or irrelevant relations. Accordingly, *we hypothesized that people who had more exposure to violence would perform equal* (based on equal relevance of age) *or worse* (based on previously documented lower performance on conventional transitive inference tasks) *at memorizing and inferring age relations relative to people who had less exposure to violence*.

We separately examined perceived neighborhood violence experienced as a bystander and direct involvement in violence, because their associations with cognitive abilities and performance could plausibly differ in weight. However, we had no a priori predictions about which kind of exposure to violence would show the highest association with our dependent variables. On the one hand, living in a dangerous neighborhood means *frequently* encountering potential dangers, and this could enhance memory and inference about dangers. On the other hand, actually being involved in violence (e.g. out of need, or due to higher levels of antisociality) increases the *relevance* of potential dangers, and this could enhance memory and inference about danger. Likewise, we examined childhood and current exposures to violence separately, again without a priori predictions regarding their relative weights. If cognitive abilities develop gradually, the association with childhood experiences may be higher. If, however, abilities adjust dynamically in response to ongoing contextual factors throughout adulthood, the association with current exposures may be higher (Frankenhuis, Panchanathan, & Nettle, 2016).

### 1.2 Training and transfer of hidden talents

If people with greater exposure to violence are indeed better able to memorize and reason about dominance relations than about abstract relations (e.g. symbols, numbers), or about less relevant, violence‐neutral social relations (e.g. minor age differences), then these enhanced abilities could potentially be leveraged in education and interventions. For instance, people could be taught mental operations (e.g. mathematics, transitive inference, syllogisms) in the context of dominance problems, which they may find more relevant and engaging than problems with more abstract or mundane content. Once they have mastered these operations in this more ecologically relevant context, they may learn to generalize them to other contents (‘near transfer’) and even to other kinds of contexts (‘far transfer’; Barnett & Ceci, 2002). By utilizing skills and social information content that are important in harsh‐dangerous environments, this instructional method works with, rather than against, social and cognitive adaptations to stress.

If this method—starting from a place of motivation and competence—succeeds, then personalized learning tools could be developed that leverage content domains (e.g. social dominance), styles of social interaction (e.g. heightened collaboration; Rogoff et al., 2017), and even executive function skills (e.g. attention shifting, working memory updating; Mittal, Griskevicius, Simpson, Sung, & Young, 2015; Young, Griskevicius, Simpson, Waters, & Mittal, 2018), that are potentially enhanced in students from high‐adversity backgrounds. Such learning tools could also enhance performance in cultural groups where formal schooling is less normative and which live in more unpredictable environments (Pope, Fagot, Meguerditchian, Washburn, & Hopkins, 2019). For example, social dominance content could serve as a starting point for adaptive learning, from which students can ‘fan out’ to other types of contents (i.e. near transfer), improving the scope of their performance.

The explosion of online learning, including at the middle and high school levels, greatly increases the opportunities for computer‐assisted instruction to be customized for specific students (Ellis et al., 2017). Two students from different backgrounds could take the same algebra course, but the course materials could, at least initially, be presented to each student in different ways. For students from high‐adversity contexts, instructional methods could utilize skills and content areas that reflect developmental adaptations to stress. If this method works, training and transfer based on hidden talents may offer a new tool to help reduce educational gaps, mitigating the pernicious cycle through which economic disadvantage translates into lasting educational disadvantage. In this way, our approach supports educational ‘equity—providing individuals with the means to thrive—rather than equality—treating everyone uniformly regardless of their specific needs’ (Moreau, Macnamara, & Hambrick, 2018, p. 4).

## 2 METHODS

The current research included two populations: an adult community sample and a college student sample. The community sample comprised people living in disadvantaged conditions for Dutch standards, some of whom have experienced chronic (i.e. prolonged, intense) stress, such as diverse exposures to violence while growing up, and others whom are currently facing an acute stressor (e.g. risk of eviction), but who have not necessarily experienced chronic stress. Members of the community sample on average had attained lower levels of education and were more likely to need governmental support in meeting their basic needs. We recruited the community sample via several organizations that help people who live in disadvantaged conditions in the Netherlands, facing such stressors as debt relief, previous incarceration, unemployment, homelessness, and neighborhood and family violence. We recruited the college student sample inside the university building via flyers and personal communication. Jointly, these samples capture a substantial range of variation in exposure to violence, from childhood experiences to present conditions.

We first conducted a pilot study in the United States with 46 participants (21 from a community sample [recruited from an employment development center in a large city] and 25 college students [from the same city]; age range 16–25), which served to refine our study design (e.g. instructions, exclusion criteria) and to obtain effect size estimates (for a recent critique of this approach, see Albers & Lakens, 2018).

We initially determined a sample size of 200 for this confirmatory study based on a power analysis via G × Power (Erdfielder, Faul, & Buchner, 1996) using a medium effect size (*f* = 0.1), 5% alpha‐level, 80% power, assuming a repeated‐measures ANOVA with a within‐between interaction (see preregistration link below). We later realized it would be better to use generalized linear mixed models. We thus switched to this analytic method before having looked at the data; therefore, this switch did not introduce bias.

We preregistered our sample size, hypotheses, and statistical analyses for the confirmatory study at the Open Science Framework: https://osf.io/c8fne/ (under Social Dominance Study). Our materials and data are accessible at: https://osf.io/3vbky/. Our study was approved by the Ethics Committee, Faculty of Social Sciences, Radboud University; CSW2014‐1310‐250.

### 2.1 Participants

Our goal was to test 200 participants: 100 students and 100 from the community sample. We tested 268 participants: 130 students and 138 community participants. We excluded 16 community participants who did not have the capacity to do the assessment (e.g. major drug use, head trauma, indicated not understanding the instructions), four who completed the dominance task but not the age task (no one completed the age task, but not the dominance task), and 17 who did not complete either task, leaving us with 101 community participants. After applying our other exclusion criteria, our final sample size comprised 199 participants: 100 students (60 females, age 18–61; *M* = 23.13, *SD* = 4.78) and 99 community participants (60 females, age 18–65; *M* = 40.14, *SD* = 11.96).

As sensitivity analyses, we also conducted our main analyses without removing the participants we over‐tested and without removing outliers (231 participants) and with all 3‐*SD* outliers removed (191 participants). We made all of the above decisions based on principled grounds, without knowing their implications for confirmatory testing. All participants received financial compensation.

### 2.2 Materials

#### 2.2.1 Stimuli

**Figure 1:**
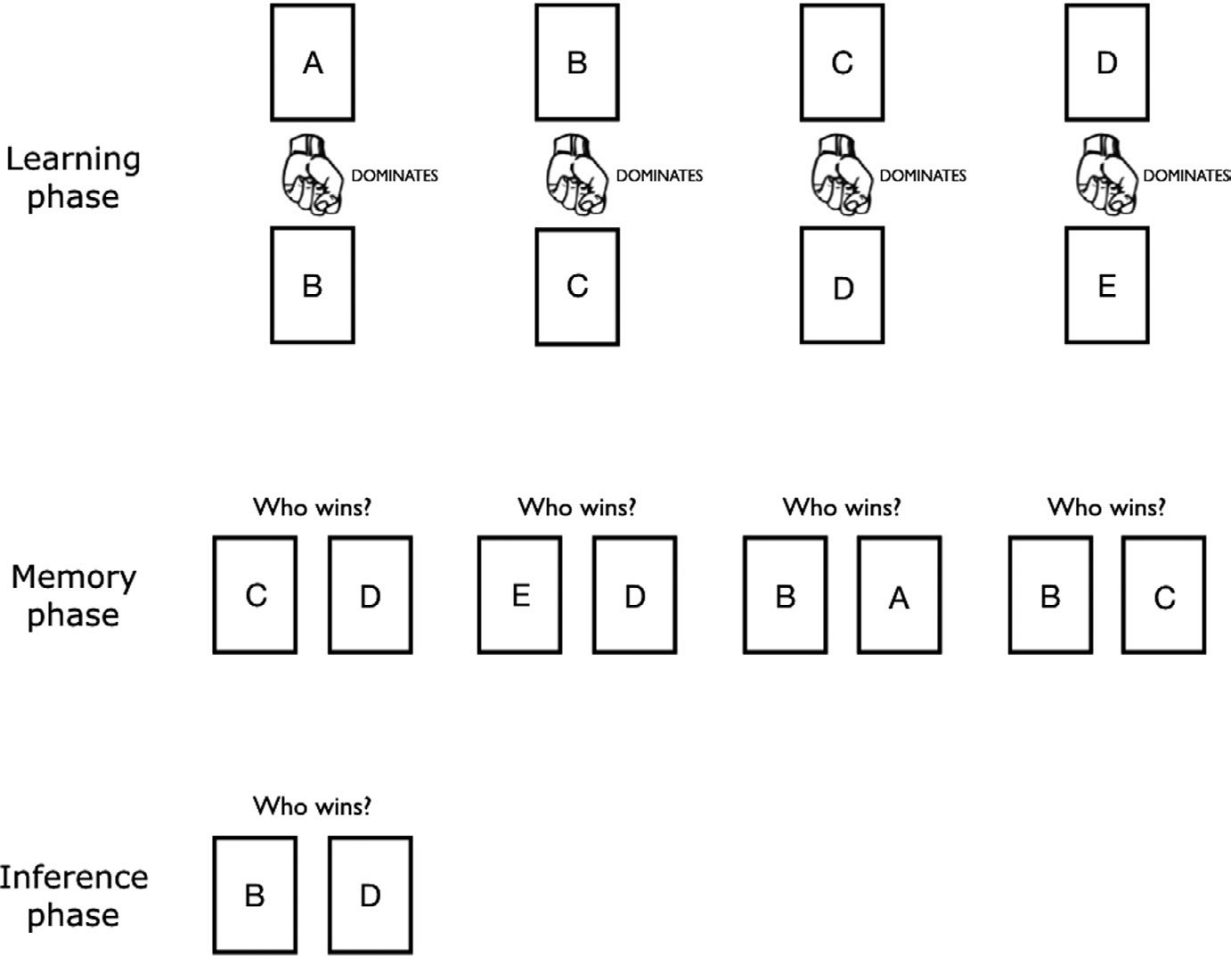
A graphical flow chart of the task structure. Here we replaced pictures of faces with letters for ease of display. The actual task given to participants contained faces. The picture (fist) and text (‘dominates,’ ‘Who wins?’) match those of the dominance task. The age task similarly displayed a picture of a ruler and the text ‘is older than’, ‘Who is older?’. In the actual stimuli, the color of text was black, except in the inference phase, where text was red to distinguish it from the learning and memory phase. For original materials, see: https://osf.io/3vbky/

In order to measure both memory and inference, we developed a novel version of the five‐item problem, which has been widely used in TI research with humans and non‐human animals (Grosenick et al., 2007; Nakamaru & Sasaki, 2003; Paz‐y‐Miño et al., 2004; Vasconcelos, 2008; Wynne, 1995). In the five‐item problem, individuals first learn a series of four overlapping pairs (A > B; B > C; C > D; D > E) and are then tested on a novel pair (B–D). We used five faces for the dominance task and five different faces for the age task; that is, a total of 10 faces. Dominance or age relations were represented using symbols: dominance by a fist, age by a ruler. The dominant or older face was always presented above the subordinate or younger face, with a symbol in between them. Figure 1 depicts a flow chart of the task structure.

The instructions for the memory phase were (original in Dutch): ‘During this task you will see two people. For each pair, we will tell you which person is more dominant [or older, in the age task]. The person placed higher on the screen is more dominant [older] than the person placed lower on the screen. Try to remember this information.’ The instructions for the inference phase stated: ‘You will now see a NEW PAIR of people. Please indicate which person is more dominant [older].’

We obtained male face stimuli and symbols through an online image search. In a pilot study, we had 14 students rate a much larger set of faces on the level of dominance for each face. We did not ask them to rate the perceived age of each face (in retrospect, it would have been good to do this). For our final stimulus set, we selected a subset of faces for which responses were mixed; that is, average ratings of these faces were in the moderate range. If people do not have strong priors about the level of dominance of a face, it will be easier to learn new information about relative dominance about this face. None of the pilot participants completed the actual study. The dimensions of the pictures were 150 (width) by 210 (height) pixels. We implemented the study in Inquisit, 2015 (4.0.8.0).

All participants completed one and the same dominance TI task and age TI task, with task order counterbalanced between participants. Whether the correct answer for the inference item on age or dominance was presented on the left or right (B–D or D–B) was also counterbalanced between participants. For all participants, the correct answer to the inference item was once presented on the left side of the screen (e.g. for dominance) and once on the right side (e.g. for age) in order to avoid simple response bias (e.g. always selecting the face on the left side) producing correct or incorrect answers to both items.

#### 2.2.2 Neighborhood violence

We measured childhood (seven items; e.g. ‘In the neighborhood where I grew up, shootings or stabbings occurred’) and current (seven items; e.g. ‘In the neighborhood where I live, physical fights are common’) exposure to neighborhood violence as a bystander using the Neighborhood Violence Scale (see Frankenhuis, Roelofs, & de Vries, 2018, for the development of this scale). The subscales are identical except in referring to childhood, that is, up to 18 years (α = 0.84), or current experiences (α = 0.87). Participants rated items on each scale from 1 to 7 (*completely agree‐disagree*). We computed an average score for each participant for each subscale, with higher scores indicating greater neighborhood violence. For both samples, the distribution of scores on both subscales was positively skewed: most scores were low, fewer were medium, and very few were high (for details, see Results section 3.2.1 section).

#### 2.2.3 Involvement in violence

We measured direct involvement in violence using a subset of four items (see preregistration) from the Youth Risk Behavior Survey (Eaton et al., 2012). One item asked about the frequency of childhood (adolescent) involvement (14–17 years) and a second item asked about the frequency of current involvement (in the last year) in a physical fight that required treatment for injuries. Participants rated these on a scale from 1 to 5 (*0–6+* *times*). The other two items asked about the frequency of childhood and current involvement in a physical fight more generally, irrespective of injuries. Participants rated these on a scale from 1 to 8 (*0–12+* *times*). These two response scales were identical to the ones used in the original Youth Risk Behavior Survey. To compute averages across scales with different numbers of response options (i.e. five or eight), we truncated 10 scores that were higher than five on the eight‐option items, assigning them a value of five (so, a score of 5 on both item types means 6+ times). Then we computed a childhood measure based on the average of the two childhood items and a current measure based on the average of the two current items.

#### 2.2.4 Exploratory instruments

We measured four constructs for exploratory analyses (for details, see preregistration). We measured parent–child relationship quality (warmth and coercion) using an abbreviated version of the Parenting Questionnaire developed by Ellis, Schlomer, Tilley, and Butler (2012). This scale included 20 items (e.g. ‘My mother pushed, grabbed, or slapped me’), and asked about the first 16 years of life, as well as current experience. We measured childhood and current basic material needs (adequacy of resources to make ends meet) using a version of the Material Needs Scale developed by Conger and Ge (1994). This scale included eight items (e.g. ‘Your family had enough money to afford the kind of clothing you all needed’), and asked about the first 16 years of life, as well as current experience. We measured perceived life expectancy (the age until which a person expects to live), using a scale of eight items that we developed (e.g. ‘Do you think you will reach the age of 70’), in the majority of community participants and about half of the student sample (we added this scale later). We analyzed these four constructs using the same kind of statistical models that we used in our confirmatory analyses.

### 2.3 Procedure

The community sample completed the study in a room of the building of the community organization from which they were recruited; students completed the study in a test‐cubicle at the university. All participants completed the questionnaires and tasks by themselves, in Dutch, and were invited to ask clarification questions to the experimenter. The community sample was tested on a 17‐inch laptop; students on a 24‐inch desktop. Stimuli were shown in the midpoint of the screen.

In the learning phase, four pairs (A > B; B > C; C > D; D > E) appeared, sequentially, on a computer screen. After each pair was shown once, for a fixed window of 5 s, participants recalled from memory, also sequentially, for each face pair, which individual is dominant or older. This learning‐memory phase repeated until participants reached the predefined threshold of correctly recalling the entire sequence twice in a row, evidencing memorization; thus, perfect performance resulted in a memory score of two rounds. The probability of reaching this threshold by chance—that is, guessing correctly eight times in a row—is very low. The number of sequences participants needed to reach this threshold indicated memory accuracy. Having reached the memory threshold, the inference phase would start. In this phase, participants answered who is dominant or older in the novel B–D pair, revealing inference accuracy (0 = incorrect; 1 = correct). After the response to a memory or inference question, the next stimulus would appear without delay. Response windows for answering the memory or inference questions were indefinite. We did not design the study to examine reaction times; hence we do not report these.

## 3 RESULTS

### 3.1 Analytic approach

We calculated generalized linear mixed models (GLMMs) with the number of memory rounds serving as the dependent variable in the first set of analyses and inference performance (correct or incorrect) serving as the dependent variable in the second set. In all analyses, (a) the target adversity measure was entered as a between‐subjects variable and (b) information type (age vs. dominance) was entered as a within‐subjects factor. We evaluated main effects and within‐between interaction effects (to allow us to formally test our main hypothesis: that the effects of adversity on the cognitive outcome variables differ as a function of information type). For memory performance, we also included task order (completed first or second) as a within‐subjects factor (as explained below). We operationalized adversity in terms of sample (community vs. student) in the group‐level analyses and in terms of exposure to or involvement in violence in the individual‐level analyses. Each model included a per‐participant intercept and no random slopes. For example, we analyzed *memory* using the following kinds of models:

$$\text{informationType}*\text{sample}+\text{order}*\text{sample}+\left(1|\text{subject}\right)$$

$$\begin{array}{c} \text{informationType}*\text{adversityMeasure}\\ \;+\text{order}*\text{adversityMeasure}+\left(1|\text{subject}\right) \end{array}$$

where the symbol * denotes both main effects and interactions. We used R to compute GLMMs with a Poisson link function to analyze memory performance (Version 3.5.0; R Development Core Team, 2018). This function matched the positively skewed distribution of the memory variables; that is, integers with many low scores and few high scores. We obtained *p*‐values based on parametric bootstrapping with type 3 sums of squares (for explanation see Luke, 2017) using the mixed () function from the afex package (Singmann, Bolker, Westfall, & Aust, 2018). For each effect, this analysis compares the full model with a model that restricts that effect to zero using a χ^2^ distribution. As inference performance was binary (correct, incorrect), we used GLMMs with a binomial link function. For example, we analyzed *inference* using the following kinds of models:

$$\text{informationType}*\text{sample}+\left(1|\text{subject}\right)$$

$$\text{informationType}*\text{adversityMeasure}+\left(1|\text{subject}\right)$$

#### 3.1.1 Error control

We evaluated two hypotheses: one about memory, and one about inference. For each, we planned to conduct five tests: one comparing the community and the student sample, and four for the continuous predictors. To control for multiple testing, we applied the sequential Holm–Bonferroni correction (Holm, 1979). We applied this correction per hypothesis, as recommended by Lakens (2016), for these five tests. We list the adjusted alpha with every *p*‐value lower than 0.05. We calculated simple slopes only for interactions with a *p*‐value lower than 0.05.

We also applied Holm–Bonferroni corrections to our four exploratory analyses, and again separately for each variable (parent–child relationship quality; childhood and current basic material needs; perceived life expectancy). For these analyses, we report only the effects that were significant after applying the correction.

### 3.2 Statistical analyses

#### 3.2.1 Descriptive statistics

We provide only descriptive, not inferential, statistics for the United States pilot study (Table 1). Although the two samples were about the same age in this pilot study, the community participants scored higher across all measures of violence exposure. The descriptive statistics further suggest that the memory and inference performance of the community participants, more than that of students, was facilitated by dominance (vs. age) content. Students generally performed better than community participants on inference and on memory for age, but not on memory for dominance. Although it appears that the community participants displayed better memory performance (Table 1), two of the three community participants that we dropped (because they did not complete both tasks) needed many rounds to complete one task. Including them would have resulted in a higher mean and larger range for this group.

**Table 1:** Statistics describing the community and student samples from the United States on childhood and current neighborhood violence, involvement in violence, and memory and inference performance

Measures	United States sample	
	Community (*n* = 18)	Students (*n* = 22)
	Mean (range)	Mean (range)
Childhood neighborhood violence	3.94 (1.00–7.00)	1.77 (1.00–4.71)
Current neighborhood violence	3.36 (1.00–5.86)	2.81 (1.29–6.00)
Childhood involvement in violence	1.78 (1.00–3.50)	1.09 (1.00–2.00)
Current involvement in violence	1.53 (1.00–3.00)	1.05 (1.00–1.50)
Memory dominance^a^	3.22 (2–6)	3.64 (2–13)
Memory age^a^	3.33 (2–6)	3.64 (2–9)
Inference dominance^b^	0.78	0.82
Inference age^b^	0.44	0.59

a Number of memory rounds;

b correct = 1, incorrect = 0, with the mean score indicating the percentage of participants (across the full sample) who answered correctly.

In the confirmatory Dutch study, the community participants also scored higher than student participants on all measures of violence exposure (Table 2). We compared the two samples using Welch's *t* test (*p*‐values) and Bayesian Independent Samples T‐Tests (Bayes Factors; BF). We computed BFs using JASP software (2018). BFs quantify the likelihood of the data conditional on Model 1 (representing H1), divided by the likelihood of the data conditional on Model 0 (representing H0). For three out of four exposure variables, BFs indicate that the data is much more likely under H1 than H0.

**Table 2:** Statistics describing the Dutch community and student samples on childhood and current neighborhood violence, involvement in violence, and memory and inference performance

Measures	Dutch sample			
	Community	Students	Tests	
	Mean (range)	Mean (range)	*T*	1‐sided BF_10_
Childhood neighborhood violence	3.74 (1.14–6.14)	2.64 (1.00–5.71)	7.11***	730,300,000
Current neighborhood violence	2.92 (1.14–6.43)	2.18 (1.14–6.00)	4.78***	9,070
Childhood involvement in violence	1.63 (1.00–4.00)	1.25 (1.00–3.00)	4.01***	490
Current involvement in violence	1.16 (1.00–3.00)	1.06 (1.00–2.00)	2.03*	2.08
Memory dominance^a^	5.34 (2–25)	3.22 (2–16)	n.a.	n.a.
Memory age^a^	5.88 (2–23)	3.13 (2–16)	n.a.	n.a.
Inference dominance^a^	0.59	0.82	n.a.	n.a.
Inference age^b^	0.59	0.77	n.a.	n.a.

Abbreviations: BF, Bayes Factors; n.a., not applicable.

a Number of memory rounds;

b correct = 1, incorrect = 0, with the mean score indicating the percentage of participants (across the full sample) who answered correctly.

* *p* < 0.05,

*** *p* < 0.001.

### 3.3 Memory

#### 3.3.1 Order effect

A GLMM with memory rounds as the dependent variable, and information type (age or dominance) and task order (first or second task) as within‐subject variables, revealed a significant main effect of task order (*χ* ^2^(1) = 58.93, *p* = 0.001). Participants needed fewer rounds to memorize the pairs in the second task than in the first task. Therefore, we controlled for order (task number) in all analyses of memory performance by including it as a main effect. We also included the interaction between the adversity measures and order because in the case of an impairment effect, those with higher adversity scores would have had more room to improve from one task to the next than those with lower adversity scores. There was no main effect of information type (*χ* ^2^(1) = 0.79, *p* = 0.38), and no interaction between information type and task number (*χ* ^2^(1) = 0.47, *p* = 0.51).

#### 3.3.2 Main analyses

The main effect of task order on memory performance was significant in all of the models (all *χ* ^2^(1) 28.69–60.10, all *p* = 0.001). There was no significant main effect of information type in any of the models (*χ* ^2^(1) = 0.008–1.86, *p* = 0.17–0.94). Students memorized both dominance and age relations better than community participants (Table 3). At an individual level, more exposure to violence (across all four indicators) was associated with lower overall memory performance. Contrary to our predictions, the main effects of childhood exposure to violence on memory were not qualified by significant interaction effects. However, consistent with our predictions, the main effects of current exposure to violence on memory were qualified by significant interaction effects.

**Table 3:** Generalized linear mixed model analysis of number of memory rounds

Between‐subject independent variable	Main effect	Interaction with information type	Interaction with order	Figure
Sample: community or student	*χ* ^2^(1) = 57.61 *p* = 0.001 *α_Holm_* = 0.0167	*χ* ^2^(1) = 0.58 *p* = 0.42	*χ* ^2^(1) = 25.44 *p* = 0.001 *α_Holm_* = 0.0125	See Table 2
Childhood neighborhood violence	*χ* ^2^(1) = 22.46 *p* = 0.001 *α_Holm_* = 0.0125	*χ* ^2^(1) = 0.59 *p* = 0.45	*χ* ^2^(1) = 11.66 *p* = 0.002 *α_Holm_* = 0.0167	Figure 2a
Current neighborhood violence	*χ* ^2^(1) = 8.30 *p* = 0.003 *α_Holm_* = 0.025	*χ* ^2^(1) = 18.20 *p* = 0.002 *α_Holm_* = 0.01	*χ* ^2^(1) = 4.03 *p* = 0.044 *α_Holm_* = 0.05	Figure 2b
Childhood involvement in violence	*χ* ^2^(1) = 18.34 *p* = 0.001 *α_Holm_* = 0.01	*χ* ^2^(1) = 0.19 *p* = 0.65	*χ* ^2^(1) = 14.87 *p* = 0.001 *α_Holm_* = 0.01	Figure 2c
Current involvement in violence	*χ* ^2^(1) = 6.90 *p* = 0.007 *α_Holm_ = *0.05	*χ* ^2^(1) = 7.56 *p* = 0.012 *α_Holm_ = *0.0125	*χ* ^2^(1) = 6.10 *p* = 0.016 *α_Holm_* = 0.025	Figure 2d

**Figure 2:**
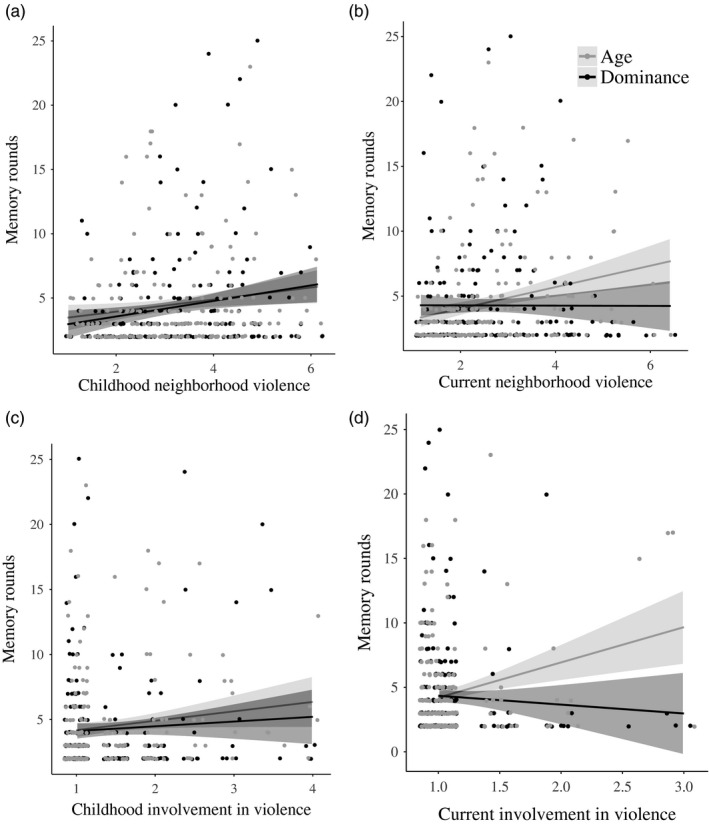
Associations between memory rounds for each information type (age or dominance) and (a) childhood neighborhood violence, (b) current neighborhood violence, (c) childhood involvement in violence, and (d) current involvement in violence. Shaded bands represent 95% confidence intervals. Participants who needed fewer memory rounds exhibited better performance. We added horizontal and vertical jitter to the dots

Simple slopes (Figure 2) revealed that current neighborhood violence positively correlated with the number of rounds needed to memorize age relations (*τ* = 0.217, *p* < 0.001, *BF*_10_ = 2,852), but not with dominance relations (*τ* = 0.020, *p* = 0.71, *BF*_01_ = 9.94). Current involvement in violence was negatively correlated with the number of rounds needed to memorize dominance relations (*τ* = −0.12, *p* = 0.048, *BF*_10_ = 2.69), but not with age relations (*τ* = 0.06, *p* = 0.37, *BF*_01_ = 5.49).

### 3.4 Inference

#### 3.4.1 Order effect

A GLMM with inference performance (correct or incorrect) as the dependent variable, and information type (age or dominance) and task order (first or second task) as within‐subject variables, revealed no significant main effects of task order (*χ* ^2^(1) = 0.18, *p* = 0.66) or information type (*χ* ^2^(1) = 0.45, *p* = 0.51), and no interaction between information type and task order (*χ* ^2^(1) = 0.33, *p* = 0.57). Therefore, we did not control for order in analyses of inference.

#### 3.4.2 Main analyses

There was no significant main effect of information type in any of the models (*χ* ^2^(1) = 0.35 – 0.50, *p* = 0.47 – 0.54). Students inferred both dominance and age relations better than community participants (Table 4). At an individual level, across all four indicators, there were no statistically significant associations between exposure to violence and overall reasoning performance. Contrary to our predictions, the main effects of childhood and current exposure to violence on inference were not qualified by significant interaction effects.

**Table 4:** Generalized linear mixed model analysis of inference performance

Between‐subject independent variable	Main effect	Interaction with information type
Sample: community or student	*χ* ^2^(1) = 18.55 *p* = 0.001 α_Holm_ = 0.01	*χ* ^2^(1) = 0.50 *p* = 0.48
Childhood neighborhood violence	*χ* ^2^(1) = 0.52 *p* = 0.50	*χ* ^2^(1) = 0.03 *p* = 0.86
Current neighborhood violence	*χ* ^2^(1) = 0.00 *p* = 0.97	*χ* ^2^(1) = 0.15 *p* = 0.70
Childhood involvement in violence	*χ* ^2^(1) = 2.46 *p* = 0.13	*χ* ^2^(1) = 1.46 *p* = 0.22
Current involvement in violence	*χ* ^2^(1) = 0.24 *p* = 0.64	*χ* ^2^(1) = 0.25 *p* = 0.62

### 3.5 Sensitivity analyses

Participant age was positively associated with the number of rounds needed to memorize age (*τ* = 0.21, *p* < 0.001, *BF*_10_ = 27,076) and dominance relations (*τ* = 0.24, *p* < 0.001, *BF*_10_ = 908). Including the main effect of age in the analyses on memory performance did not change qualitative results from the main analyses. Welch's *t* tests showed no significant gender difference on age memory (*t* = −1.01, *p* = 0.31) or dominance memory (*t* = −0.28, *p* = 0.78).

#### 3.5.1 More liberal inclusion criteria (231 participants)

Results did not differ qualitatively from our main analyses.

#### 3.5.2 More conservative inclusion criteria (191 participants)

Results did not differ qualitatively from our main analyses in most cases. However, there was no significant main effect of current involvement in violence on memory rounds (*χ* ^2^(1) = 1.70, *p* = 0.20). Additionally, the interaction between sample and information type on memory, though still non‐significant, resulted in a lower *p*‐value (*χ* ^2^(1) = 3.60, *p* = 0.060).

#### 3.5.3 Correcting for sample

We did not include sample in our preregistered analyses of the continuous predictors, because we expected substantial overlap between variation in sample and these predictors. Including the main effect of sample in the questionnaire analyses did not change most results. Notably, theoretically relevant interaction effects remained significant. However, the main effects of current neighborhood violence (*χ* ^2^(1) = 4.00, *p* = 0.052) and current involvement in violence (*χ* ^2^(1) = 5.41, *p* = 0.0251, *α_Holm_* = 0.025) became non‐significant.

### 3.6 Auxiliary hypotheses

There was a significant main effect of childhood basic financial needs on overall memory performance (*χ* ^2^(1) = 9.08, *p* = 0.006, *α_Holm_* = 0.0167). Those whose families were better able to pay for their basic financial needs needed fewer rounds to memorize the relations. The same was true for current basic financial needs (*χ* ^2^(1) = 24.15, *p* = 0.001, *α_Holm_* = 0.0125). None of the exploratory questionnaires had significant main or interaction effects on inference performance.

### 3.7 Exploring the cognitive measures

A GLM with a Poisson link function predicting one type of memory performance from the other and controlling for task order showed either a non‐significant or a significant relation, depending on whether age (*Estimate* = 0.019, *SE* = 0.009, *p* = 0.039) or dominance (*Estimate* = 0.016, *SE* = 0.008, *p* = 0.053) was the predictor.

Participants who correctly answered the age inference question were more likely to correctly answer the dominance inference question (*χ* ^2^(1) = 10.02, *p* = 0.002, *BF*_10_ = 38.33).

Logistic regressions indicated that the number of rounds needed to memorize dominance relations was not associated with dominance inference performance (*Estimate* = −0.05, *p* = 0.20). However, the number of rounds needed to memorize age relations was significantly associated with age inference performance (*Estimate* = −0.09, *p* = 0.03).

## 4 DISCUSSION

Students memorized and inferred both dominance and age relations better than community participants. Further, more childhood and current neighborhood violence, and involvement in violence, were associated with lower memory performance, but not with reasoning performance. However, the main effects of current neighborhood violence and involvement in violence on memory performance were qualified by significant interaction effects. Specifically, more current neighborhood violence was associated with worse memory for age relations, but not with memory for dominance relations. More current involvement in violence was associated with *better* memory for dominance relations, but not with memory for age relations. This pattern of equal or enhanced performance despite greater exposure to adversity is striking when considering the developmental literature, which has nearly exclusively revealed deficits in the social and cognitive abilities of people who grow up in harsh conditions (but see Ellis et al., 2017; Frankenhuis & de Weerth, 2013).

Our main effects are consistent with the deficit model. However, our interaction effects on current exposure to violence are consistent with the specialization hypothesis. Only the specialization hypothesis predicts that people from harsh environments show equal or even enhanced performance on tasks matching recurrent problems in those environments (Ellis et al., 2017; Frankenhuis & de Weerth, 2013). We speculate that these interactions reflect a blend of impairment and specialization processes operating in concert, where impairment generally lowers performance, but specialization enhances it, specifically for relevant contents, such as social dominance. Notably, we find interaction effects only for current experiences, not for childhood experiences; there, we find only impairment. This difference suggests that, in our study, intact or enhanced performance despite greater adversity is more likely to result from a dynamic adjustment of cognition in response to current contextual factors than from gradual tailoring of cognitive abilities over the course of childhood.

### 4.1 Implications for training and transfer

An open and interesting question is what mechanisms explain performance in our study. We speculate that the relevance of social dominance content increased the level of interest, hence the attention and motivation, more so for participants who currently live in hostile conditions. Emotion research shows that military veterans reasoned better about combat‐related syllogisms than formally identical syllogisms with neutral contents (Blanchette & Caparos, 2013). This advantage could not be attributed to variation in expertise about combat. The authors theorize: ‘When the affective reaction is relevant to the semantic contents reasoned about, emotions may have a positive impact on reasoning and this effect may be mediated by utility’ (p. 412).

Although we do not find enhancement of reasoning, social dominance content may elicit stronger emotions in people who are exposed to hostile conditions (given the greater relevance and higher utility of this content), thus enhancing their memory performance. Such a process would be consistent with studies showing enhanced performance in individuals from unpredictable environments only under conditions of primed economic hardship or uncertainty (Dang et al., 2016; Mittal et al., 2015; Young et al., 2018). In addition to enhancing attention and motivation, research on ‘everyday’ (or ‘street’) mathematics shows that people also employ different cognitive strategies in‐context when solving problems with ecologically relevant content, compared with more abstract problems in formal school settings (Banerjee et al., 2017; Schliemann & Carraher, 2002). Future research could examine whether participants who are exposed to high levels of violence use different strategies when solving memory and inference problems involving social dominance versus age content. As noted above, understanding what contents and formats improve cognitive abilities in individuals who have been exposed to significant social and economic adversity provides a potential lever for enhancing their educational outcomes (Ellis et al., 2017). Our findings suggest that people who are currently exposed to or involved in violence learn differences between young men in dominance position more readily than differences between young men in age. This finding is important because it suggests that individuals living under harsh conditions may be able to hold their own, or even excel, when solving problems in which the content is adaptively relevant to their lives. The next critical research question is the transfer of such abilities across contexts and contents. For instance, we could first ask students to memorize relations that involve social dominance content, then less‐relevant yet social content (e.g. age), then non‐social content (e.g. objects), and finally abstract content (e.g. symbols). Similarly, the format of tasks can be gradually modified (e.g. from pictures, to words, to formal symbols). The importance of ecologically valid content and testing conditions has been demonstrated in past research on the mathematical ability of children from high‐adversity backgrounds (Banerjee et al., 2017; Schliemann & Carraher, 2002; Sternberg, Lipka, Newman, Wildfeuer, & Grigorenko, 2006).

Such an approach could not only benefit students who struggle in traditional school settings, but also gifted students who excel relative to their peers. A recent review of effective curriculum interventions concludes that gifted students from low‐income backgrounds have ‘a pragmatic outlook that encourages their preference for concreteness in learning, for practical applications of knowledge in their world and for examples that both come from and harken back to their world’ (VanTassel‐Baska, 2018, p. 69). Using students’ stress‐adapted skills as a starting point to gain mastery in a given domain, and then transferring these capacities by guiding them towards other kinds of contents and formats, could be a promising avenue for improving academic outcomes.

More generally, we need to know how to optimize educational settings for different populations, especially for marginalized groups, and for different age groups (Ellis et al., 2017; Markant, Ackerman, Nussenbaum, & Amso, 2016). We can examine variations in curricular content (e.g. abstract vs. social), format (e.g. pictures vs. symbols), information delivery (e.g. static books vs. dynamic touch screens), and instructional practices (e.g. sitting vs. moving around in the classroom). Such an approach fits with work in other populations that struggle in conventional school settings, such as children with attention‐deficit hyperactivity disorder (ADHD). Recent studies show that the performance of these children is enhanced, more than that of controls, if they are allowed to learn while moving around (Hartanto, Krafft, Iosif, & Schweitzer, 2016; Sarver, Rapport, Kofler, Raiker, & Friedman, 2015). What is critically needed is more research that delineates the contexts that maximize performance of stress‐adapted children and youth (Ellis et al., 2017; Frankenhuis & de Weerth, 2013; Goudeau & Croizet, 2017; Richardson, Castellano, Stone, & Sanning, 2016). In the words of Barbara Rogoff et al.: ‘A challenge for future research is looking for strengths in all populations and designing learning situations and assessments in ways that build on and build toward the strengths of all’ (2017, p. 885). We thus view stress‐adapted memory for ecologically relevant information, such as social dominance, as a starting point for further learning. Working in concert with these abilities can help to improve learning outcomes and hence reduce educational inequalities and its sequelae for socioeconomic position and health. But before we design interventions, we need solid scientific foundations.

### 4.2 Limitations

Our preregistered study evaluates a diverse group of participants; however, it also has limitations. First, the test setting differed, inevitably, between our student and community sample. Second, testing community participants in a computerized setting, rather than in a more hands‐on, real‐world, practical setting, could have lowered their performance (Ellis et al., 2017). Third, we obtained only two memory and inference scores per participant; more measurements would have allowed for more precise estimates of ability. This point applies more so to our dichotomous measure of inference than to our continuous measure of memory. Fourth, we measured involvement in violence using a scale that captures only serious involvement in violence, resulting in many low scores. Future work should include a scale that is more sensitive to individual differences in less serious involvement in violence, such as verbal threats (e.g. a version of the Aggression Scale; Orpinas & Frankowski, 2001). Fifth, the correlation between age and dominance memory performance was lower than we expected. This could reflect a measurement issue or genuine differences in the ability to solve our task depending on its content. Richardson (1991) found similarly low correlations between socially more or less meaningful versions of the Raven Progressive Matrices task. Sixth, our research design was correlational. Therefore, we are not able to rule out the possibility that genetic variation has contributed to the empirical relations we have documented. Despite these limitations, we consider our study more breakthrough than incremental, because it is one of the first studies to systematically investigate, and offer tentative evidence for, enhanced skills and abilities in people who are exposed to higher levels of adversity.

## Data Availability

The materials and data of this work are accessible at: https://osf.io/3vbky/.
